# Study of Possible Mechanisms Involved in the Inhibitory Effects of Coumarin Derivatives on Neutrophil Activity

**DOI:** 10.1155/2013/136570

**Published:** 2013-11-17

**Authors:** Katarína Drábiková, Tomáš Perečko, Radomír Nosál', Juraj Harmatha, Jan Šmidrkal, Viera Jančinová

**Affiliations:** ^1^Institute of Experimental Pharmacology and Toxicology Slovak Academy of Sciences, Dúbravská cesta 9, 841 04 Bratislava, Slovakia; ^2^Institute of Organic Chemistry and Biochemistry, Academy of Sciences of the Czech Republic, v. v. i., Flemingovo náměsti 2, 161 10 Prague, Czech Republic; ^3^Institute of Chemical Technology Prague, Technická 5, 166 28 Prague, Czech Republic

## Abstract

To specify the site of action of the synthetic coumarin derivatives 7-hydroxy-3-(4′-hydroxyphenyl) coumarin (HHC) and 7-hydroxy-3-(4′-hydroxyphenyl) dihydrocoumarin (HHDC), we evaluated their effects on extra- and intracellular reactive oxygen species (ROS) formation in phorbol-myristate-13-acetate (PMA) stimulated human neutrophils. We studied also the effects of HHC and HHDC on possible molecular mechanisms which participate in the activation of NADPH oxidase, that is, on PKC activity, on phosphorylation of some PKC isoforms (**α**, **β**II, and **δ**), and on phosphorylation of the NADPH oxidase subunit p40^*phox*^. Without affecting cytotoxicity, both coumarines tested were effective inhibitors/scavengers of ROS produced by neutrophils on extracellular level. HHC markedly diminished oxidant production and also, intracellularly, decreased PKC activity and partly phosphorylation of PKC**α**, **β**II. On the other hand, we did not observe any effect of coumarin derivatives on phosphorylation of PKC**δ** and on phosphorylation of the NADPH oxidase subunit p40^*phox*^, which were suggested to be involved in the PMA-dependent intracellular activation process. In agreement with our previous findings, we assume that the different molecular structures of HHC and HHDC with their different physicochemical and free radical scavenging characteristics are responsible for their diverse effects on the parameters tested.

## 1. Introduction

Neutrophils are key cells of the first line of defense, but they are also considered potent inflammatory cells causing tissue damage. Thus the ability of compounds which prevent extensive and potentially dangerous activation of neutrophils has been proposed as an important injury-limiting way. Coumarins belong to the group of plant-derived polyphenolic compounds possessing broad biochemical and pharmacological effects, like anti-HIV, anti-inflammatory, antioxidant, antibacterial, anticoagulant, and anticancer activities [1–4]. Over the last years, natural as well as synthetic coumarins were extensively studied and many of them are considered attractive candidates in therapeutic development. 

Production of reactive oxygen species (ROS) in neutrophils and other phagocytic cells is linked to the activation of NADPH oxidase, a multiprotein enzyme complex, which plays an essential role in innate immunity. Yet excessive ROS generation by phagocytes is involved in tissue injury associated with a number of chronic inflammatory diseases [5–7]. In resting cells, NADPH oxidase is inactive and its components are distributed between the cytosol and membranes. When cells are activated, the cytosolic components (p40^*phox*^, p47^*phox*^, p67^*phox*^, and Rac2) migrate to the membranes, where they associate with the membrane-bound component (flavocytochrome b558) to assemble the catalytically active NADPH oxidase, resulting in the delivery of ROS into the extracellular environment or inside into the phagocytic vesicle [8–10]. NADPH oxidase is also activated within specific intracellular compartments, leading to an intracellular ROS production which may have a direct role in signal transduction. It was reported that the cytosolic component of the NADPH oxidase, p40^*phox*^, associates with cytochrome b, especially in intracellular membranes [10–13]. Localisation of neutrophil ROS production and its possible regulation thus play an important role in the development of effective treatments to control the damage associated with chronic inflammation [8, 14]. 

Protein kinase C (PKC) has been shown to activate NADPH oxidase in several types of cells, such as phagocytes, cardiomyocytes, aortic endothelial cells, vascular smooth muscle cells, and renal mesangial cells [15, 16]. Various PKC isoforms have been shown to stimulate superoxide production, including *α*, *β*, *δ*, *ε*, and *ζ* [17, 18]. Neutrophils are strongly activated by PKC activating phorbol esters, which together with the expression of several PKC isoforms in neutrophils suggest a role for PKC in neutrophil functions [19]. Human neutrophils contain multiple isotypes of PKC, including Ca^2+^/DG-dependent isotypes-*α*; alternatively spliced *β*I and *β*II; Ca^2+^-independent/DG-dependent isotype *δ*; and phosphatidylserine-dependent Ca^2+^/DG-independent *ξ* [19, 20]. In cell-free systems, PKC*α*, *β*, and *δ* are implicated as regulators of NADPH oxidase and superoxide generation [21]. Moreover, PKC*δ* has been suggested to be responsible for intracellular ROS generation in PMA activated neutrophils [22].

Previously, we compared the effect of 7-hydroxy-3-(4′-hydroxyphenyl) coumarin (HHC) and 7-hydroxy-3-(4′-hydroxyphenyl) dihydrocoumarin (HHDC) on stimulated phagocyte functions with their physicochemical characteristics and free radical scavenging activities in chemical assays [23].

**Figure 1:**
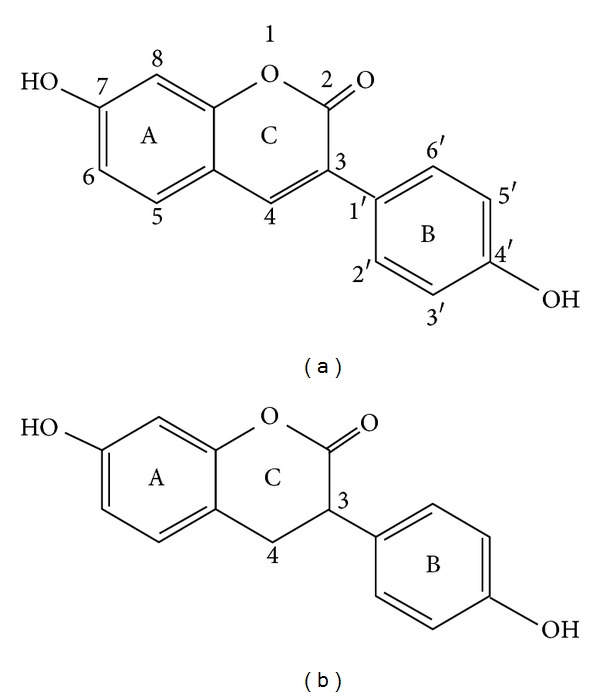
Chemical structure of synthetic phenylcoumarin derivatives related to the natural 7-hydroxycoumarin (umbelliferon). (a) 7-Hydroxy-3-(4′-hydroxyphenyl) coumarin (HHC). (b) 7-Hydroxy-3-(4′-hydroxyphenyl)-3,4-dihydrocoumarin (HHDC).

To specify the site of action of the synthetic coumarin derivatives HHC and HHDC (Figure 1), we evaluated their effects on extra- and intracellular ROS formation in PMA stimulated human neutrophils. We also studied the effects of HHC and HHDC on possible molecular mechanisms which participate in the activation of NADPH oxidase: on PKC activity, phosphorylation of some PKC isoforms (*α*, *β*II, and *δ*), and on phosphorylation of the subunit p40^*phox*^.

## 2. Material and Method

Phorbol-myristate-13-acetate (PMA), luminol (5-amino-2,3-dihydro-1,4-phthalazinedione), isoluminol (6-amino-2,3-dihydro-1,4-phthalazinedione), superoxide dismutase, dextran (average MW 464.000), and protease inhibitor cocktail: 104 mmol/L AEBSF [4-(2-aminoethyl) benzenesulfonyl fluoride hydrochloride], 0.085 mmol/L aprotinin, 1.53 mmol/L bestatin hydrochloride, 1.40 mmol/L E-64 [N-(trans-epoxysuccinyl)-L-leucine 4-guanidinobutylamide], 1.90 mmol/L leupeptin hemisulfate salt, and 4.22 mmol/L pepstatin in DMSO, were purchased from Sigma-Aldrich Chemie (Deisenhofen, Germany), horse radish peroxidase (HRP) and catalase from Merck (Darmstadt, Germany), and Lymphoprep (density 1.077 g/mL) from Nycomed Pharma AS (Oslo, Norway).

HHC and HHDC were kindly provided by Dr. Juraj Harmatha from the Institute of Organic Chemistry and Biochemistry, AV ČR, Prague, Czech Republic, and Professor Jan Šmidrkal from the Institute of Chemical Technology, Department of Dairy and Fat Technology, AV ČR, Prague, Czech Republic. 

HHC (1.27 mg) was dissolved in a mixture of 20 *μ*L 1 NaOH and 980 *μ*L Tyrode solution (Tyrode solution consisted of 136.9 mmol/L NaCl, 2.7 mmol/L KCl, 11.9 mmol/L NaHCO_3_, 0.4 mmol/L NaH_2_PO_4_ · 2H_2_O, 1 mmol/L MgCl_2_ · 6H_2_O, and 5.6 mmol/L glucose, pH 7.4). The stock solution (5 mmol/L) was further diluted with Tyrode solution to give HHC samples, concentrations 0.01–100 *μ*mol/L. The corresponding final concentrations of NaOH were 0.4–400 *μ*mol/L; at these concentrations, the solvent agent alone did not reduce the activity and viability of neutrophils.

All other chemicals of analytical grade were from available commercial sources. 

This work was approved by the Local Ethic Committee, Institute of Experimental Pharmacology and Toxicology SAS.

### 2.1. Blood Collection and Isolation of Human Neutrophils

Fresh blood was taken at the blood bank from healthy volunteers (men, aged 20 to 50 years) by antecubital venipuncture and was immediately mixed with 3.8% v/w trisodium citrate, in the ratio of 9 mL of blood to 1 mL of citrate, in polypropylene centrifugation tubes. Erythrocytes were allowed to sediment in 3% dextran solution (1 ×g, 25 min, 22°C) and neutrophils were separated by gradient centrifugation on Lymphoprep (500 ×g, 30 min, 22°C). After hypotonic lysis of contaminating erythrocytes, neutrophils were washed and resuspended in calcium- and magnesium-free phosphate buffer saline in mmol/L: 137 NaCl, 2.7 KCl, 8.1 Na_2_HPO_4_, 1.5 KH_2_PO_4_, and pH 7.4, at final concentration of 1 × 10^7^ cells/mL. The obtained cell suspension contained more than 96% viable cells, as evaluated by trypan blue exclusion [24, 25]. Prior to neutrophil activation studies, the cells were resuspended in phosphate buffer saline (PBS) in mmol/L: 137 NaCl, 2.7 KCl, 8.1 Na_2_HPO_4_, 1.5 KH_2_PO_4_, 1.8 CaCl_2_, 0.5 MgCl_2_ · 6H_2_O, and pH 7.4.

### 2.2. Chemiluminescence Assay of Isolated Neutrophils

The effect of HHC or HHDC (0.01, 0.1, 1, 10, and 100 *μ*mol/L) on extra- and intracellular ROS production was measured in unstimulated and PMA (0.05 *μ*mol/L) stimulated neutrophils (5 × 10^5^/sample) by isoluminol-/luminol-enhanced chemiluminescence (CL). Extracellular CL was determined in the system containing isoluminol (5 *μ*mol/L) and HRP (8 U/mL). Intracellular CL was measured with luminol (5 *μ*mol/L) in the presence of the extracellular scavengers superoxide dismutase (100 U/mL) and catalase (2 000 U/mL). CL was evaluated in a microplate luminometer Immunotech LM-01T (Czech Republic) at 37°C. Data were based on integral values of CL over 1800 s, (RLU × s; RLU: relative light units) [26, 27].

### 2.3. Chemiluminescence Assay in Cell-Free System

In the cell-free system, luminol, horseradish peroxidase, and hydrogen peroxide were used for chemiluminescence generation [28]. The 200 *μ*L samples consisted of 50 *μ*L aliquots: HRP (2 U/mL), luminol (10 *μ*mol/L), HHC, or HHDC solutions in the concentrations of 1, 10, or 100 *μ*mnol/L. The reaction was initiated by adding hydrogen peroxide at the final concentration of 100 *μ*mol/L. The chemiluminescence responses were measured for 10 minutes at 37°C in a microplate luminometer Immunotech LM-01T (Czech Republic).

### 2.4. PKC Activity Assay

PKC activity in the cytosol was detected by the modified method of Varga et al. [29]. Isolated human neutrophils (5 × 10^5^/mL) were incubated 30 min with HHC (1, 10, and 100 *μ*mol/L) or HHDC (1, 10, and 100 *μ*mol/L) at 37°C. Neutrophils were then stimulated with PMA (0.15 *μ*mol/L, final concentration) at 37°C for 3 min. The reaction was stopped by addition of 10 vol of ice-cold PBS. After centrifugation (500 ×g, 4°C, 10 min) the cells were resuspended in sample buffer (20 mmol/L TRIS-HCl, 5 mmol/L EDTA, 1% Triton, 10% glycerol, and protease inhibitor cocktail), sonicated on ice, and centrifuged (14 000 ×g, 5 min, 4°C). The cytosolic fractions were transferred to a prechilled 1.5 mL microcentrifuge tube and stored at –70°C. Protein content of cytosol fraction was measured using Bradford Dye Reagent Detection Kit (Bio-Rad, USA). PKC activity was measured with a Nonradioactive Protein Kinase Assay kit (Assay Designs, Ann Arbor, MI, USA), which is based on a solid-phase enzyme-linked immunoabsorbent assay (ELISA), utilising a specific synthetic peptide as substrate for PKC and a polyclonal antibody recognizing the phosphorylated form of the substrate. The assay is developed with tetramethylbenzidine substrate and a colour develops in proportion to PKC phosphotransferase activity. The colour development was stopped with acid stop solution and the intensity of the colour was measured in a microplate reader at 450 nm. The data were expressed as relative kinase activity per 1 mg of protein. 

### 2.5. Western-Blot Analysis

Phosphorylation of PKC isoenzymes *α*, *β*II, and *δ*, as well as NADPH oxidase subunit p40^*phox*^, was detected using the method described by Jančinová et al. [24, 27].

The suspension of isolated neutrophils (100 *μ*L) containing 5 × 10^6^ cells was preincubated at 37°C for 60 seconds with different concentrations of HHC or HHDC (10, 100 *μ*mol/L) prior to addition of PMA (0.15 *μ*mol/L). Incubation with PMA (5 min) was stopped by using a solubilisation buffer (20 mmol/L Tris-HCl, 5 mmol/L EDTA, 1% Triton, 10% glycerol, 10 mmol/L NaF, 1 mmol/L sodium orthovanadate, 208 *μ*mol/L AEBF, 0.17 *μ*mol/L aprotinin, 8 *μ*mol/L bestatin, 2.8 *μ*mol/L E-64, 4 *μ*mol/L leupeptin, and pH 7.4). The suspension was sonicated at 4°C for 20 minutes and centrifuged at 14 000 ×g at 4°C for 5 min to remove unbroken cells. The supernatant was taken for measuring total protein by using Bradford Dye Reagent Detection Kit from Bio-Rad and for blotting assay. The supernatant for blotting assay was boiled for 5 min with sample buffer (50 mmol/L Tris-HCl, 2% SDS, 7.5% glycerol, 2.5% mercaptoethanol, 0.01% bromophenol blue, and pH 6.8) and samples were loaded on 9.8% SDS polyacrylamide gels. Proteins (20 *μ*g per lane) were separated by electrophoresis and transferred to Immobilon-P Transfer Membrane (Millipore Corp., USA). From the two strips taken, one was detected for PKC*α*, *β*II (area between 60 and 100 kD) and the second for detection of *β*-actin (30–60 kD), which represented the internal control. Membrane strips were blocked for 60 min with 1% bovine serum albumin in Tris buffered saline (TBS: 20 mmol/L Tris-HCl, 154 mmol/L NaCl, 0.05% Tween-20, and pH 7.5). This was then followed by 60 min incubation in the presence of the primary antibodies: anti-phospho-PKC-*α*, *β*II (Thr638/641) (1 : 5 000) or *β*-actin (rabbit anti-human, 1 : 4 000) (Cell Signaling Technology, Danvers, MA, USA). The membranes were subsequently washed six times with TBS and incubated 60 min with the secondary antibody conjugated to horseradish peroxidase (anti-rabbit from donkey, 1 : 5 000, GE Healthcare Life Sciences, Little Chalfont, UK), and the activity of horseradish peroxidase of the bands corresponding to individual PKC isoforms was visualised using Enhanced Chemiluminescence Western Blotting Detection Reagents (GE Healthcare Life Sciences, Little Chalfont, UK).

NADPH oxidase subunit p40^*phox*^ and PKC isoenzyme *δ* were detected in the same blots after their washing and incubation with 10x diluted stripping buffer (Reblot Plus Mild Solution, Millipore, Temecula, CA, USA) for 15 min and washed overnight in diluent buffer (1% bovine serum albumin in Tris buffered saline). This was followed by 60 min incubation in the presence of primary antibodies: anti-phospho-p40^*phox*^ (T154) (1 : 5 000) or PKC*δ* (Thr505) (1 : 1 000) (Cell Signaling Technology, Danvers, MA, USA). The membranes were subsequently washed six times with TBS and incubated 60 min with the secondary antibody conjugated to horseradish peroxidase (anti-rabbit from donkey, 1 : 5 000, GE Healthcare Life Sciences, Little Chalfont, UK), and the activity of horseradish peroxidase of the bands corresponding to the individual NADPH oxidase subunit or to PKC isoform was visualised using Enhanced Chemiluminescence Western Blotting Detection Reagents (GE Healthcare Life Sciences, Little Chalfont, UK). Autoradiogram bands were quantified using the Image J programme. The optical density of each PKC or NADPH oxidase band was corrected by the optical density of the corresponding *β*-actin band [24, 27].

### 2.6. Measurement of Cytotoxicity

The cytotoxic effect of HHC and HHDC was evaluated by means of ATP liberation by the luciferin-luciferase chemiluminescence. The neutrophil suspension (30 *μ*L; 30 000 cells/sample) and 20 *μ*L of Tyrode's solution were incubated with 50 *μ*L of HHC or HHDC (1–100 *μ*mol/L) for 15 min at 37°C. Ten microliters of a mixture of luciferin (1.6 *μ*g/sample) and luciferase (45 000 U/sample) was added, and the chemiluminescence was recorded for 60 s. In each experiment, the chemiluminescence of ATP standards (1–500 nmol/L) was measured and the concentrations of ATP in samples were calculated from the calibration curve. The total ATP content was assessed immediately after sonication of neutrophils for 10 s [24, 27].

### 2.7. Statistical Analyses

All values are given as means of 4–8 experiments ± SEM. Statistical significance of differences between means was established by Student's *t*-test and *P* values below 0.05 were considered statistically significant.

## 3. Results

### 3.1. Effects of HHC and HHDC on ATP Liberation

To determine cytotoxicity of HHC and HHDC, we tested their effect on ATP liberation (Table 1). In the concentrations of 1, 10, and 100 *μ*mol/L, these compounds did not increase spontaneous ATP liberation (12.6 ± 0.9 nmol/L ATP), representing 7.6% of the total ATP content (158.7 ± 12.1 nmol/L ATP), as determined immediately after complete neutrophil destruction. The results indicate that treatment with increasing concentrations of HHC and HHDC did not cause neutrophil damage. 

### 3.2. Effects of HHC and HHDC on Extra- and Intracellular Chemiluminescence of Neutrophils

**Figure 2:**
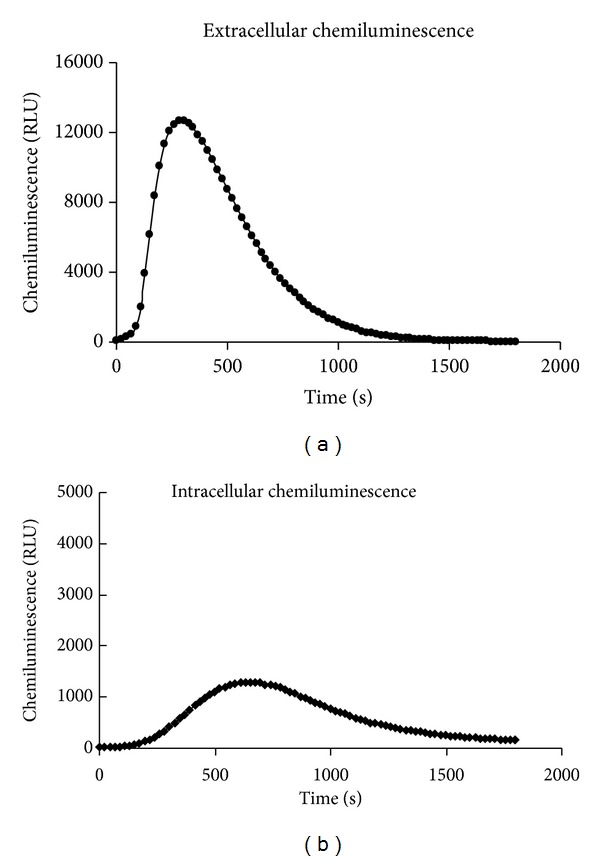
Kinetic curves of extra- and intracellular ROS formation in PMA stimulated human neutrophils. Human isolated neutrophils (5 × 10^5^/sample) were stimulated with PMA (0.05 *μ*mol/L) at 37°C. Extra- and intracellular ROS production were measured by isoluminol-/luminol-enhanced chemiluminescence over 1800 s. Kinetic curves are representative of 6 donors. RLU: relative light units.

For activation of isolated human neutrophils, the soluble stimulus PMA was used, which bypasses receptors and activates NADPH oxidase via redistribution of PKC and phosphorylation of several proteins. As mentioned above, PMA is useful in investigating signal transduction pathways leading to NADPH-oxidase activation in plasma (extracellular) and granule membranes (intracellular) [10, 11].  Figure 2 demonstrates kinetics of extra- and intracellular ROS generation in isolated human neutrophils after PMA stimulation. The extracellular ROS generation was much more intensive and reached the maximum sooner than did the intracellular ROS generation. The ratio between extra- and intracellular ROS generation was approximately 10 : 1 (12 696 RLU : 1 295 RLU). 

**Figure 3:**
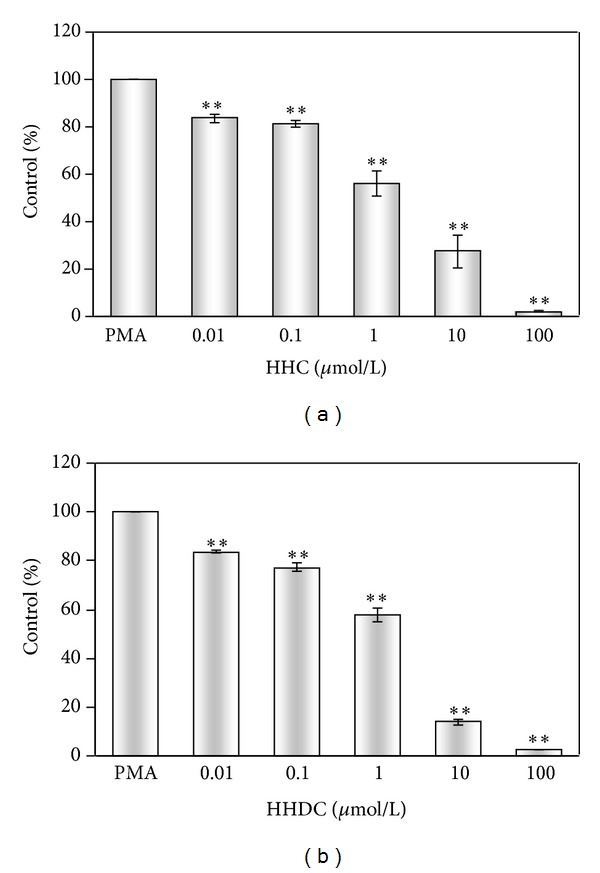
Effect of HHC and HHDC on PMA stimulated extracellular chemiluminescence of human neutrophils. Human isolated neutrophils (5 × 10^5^/sample) were stimulated with PMA (0.05 *μ*mol/L) at 37°C in the presence of HHC or HHDC (0.01–100 *μ*mol/L). Extracellular ROS production was measured in the presence of HRP by isoluminol-enhanced chemiluminescence over 1800 s. The values were calculated as percentage of stimulated (PMA) control, on the basis of integrated values of chemiluminescence over 1800 s. Control value given in RLU × seconds was 5.8 × 10^6^ ± 1.4 × 10^6^. Mean ± SEM, *n* = 6. **P < 0.01 as compared with the control (PMA) in the absence of the substances tested.

Figure 3 shows that both compounds, HHC and HHDC, in the concentration scale of 0.01–100 *μ*mol/L, decreased significantly extracellular ROS production, as measured by chemiluminescence. The concentrations of HHC and HHDC producing 50% inhibition (IC_50_) of control extracellular chemiluminescence were 1.04 ± 0.20 *μ*mol/L and 1.01 ± 0.13 *μ*mol/L, respectively (Table 2). 

**Figure 4:**
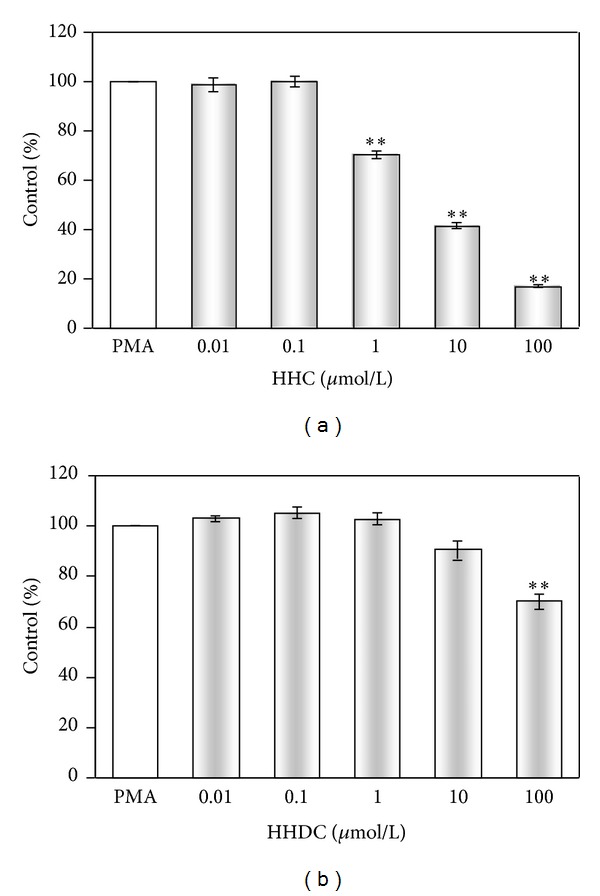
Effect of HHC and HHDC on PMA stimulated intracellular chemiluminescence of human neutrophils. Human isolated neutrophils (5 × 10^5^/sample) were stimulated with PMA (0.05 *μ*mol/L) at 37°C in the presence of HHC or HHDC (0.01–100 *μ*mol/L). Intracellular ROS production was measured in the presence of superoxide dismutase and catalase by luminol-enhanced chemiluminescence over 1800 s. The values were calculated as percentage of stimulated (PMA) control, on the basis of integrated values of chemiluminescence over 1800 s. Control value given in RLU × seconds was 5 × 10^5^ ± 0.5 × 10^4^. Mean ± SEM, *n* = 6. **P < 0.01, *P < 0.05 as compared with the control (PMA) in the absence of the substances tested.

While HHC in the concentration scale of 0.01–100 *μ*mol/L decreased significantly intracellular ROS production (IC_50_: 5.24 ± 0.57 *μ*mol/L), HHDC was slightly effective only in the highest concentration tested (Figure 4 and Table 2). 

### 3.3. Effects of HHC and HHDC on Cell-Free Chemiluminescence System

Further we tested the participation of direct antioxidant activity of HHC and HHDC in a cell-free CL system consisting of luminol, horseradish peroxidase, and hydrogen peroxide. Our results showed an effective inhibition of chemiluminescence generated by cell-free system with HHC (IC_50_: 0.59 ± 0.01) and HHDC (IC_50_: 2.47 ± 0.04) (Table 2). 

### 3.4. Effects of HHC and HHDC on PKC Activity and on the Phosphorylation of the PKC Isoforms (PKC*α*, *β*II, and *δ*)

**Figure 5:**
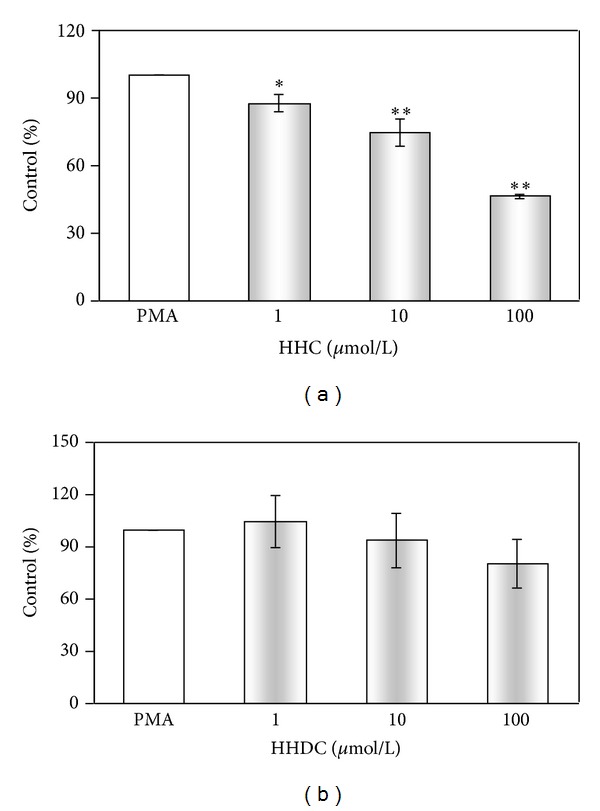
Effect of HHC and HHDC on PKC activity. Human isolated neutrophils (5 × 10^5^/mL) were incubated for 30 min with HHC or HHDC (1, 10, and 100 *μ*mol/L) and stimulated with PMA (0.15 *μ*mol/L) at 37°C for 3 min. PKC activity was measured by ELISA kit in the supernatant of cell lysate. The values were calculated as percentage of stimulated (PMA) control. Control value given as relative kinase activity (450 nm) per 1 mg of protein was 9 708 ± 1 168. Mean ± SEM, *n* = 8. **P < 0.01, *P < 0.05 as compared with the control (PMA) in the absence of the substances tested.

Stimulation of neutrophils with phorbol esters activates the PKC isoforms *α*, *β*II, and *δ*, which are involved in the oxidative burst as key activators of NADPH oxidase. First we analysed whether modulation of PKC activity by HHC or HHDC might mediate their inhibitory effect on PMA stimulated ROS production. HHC in the concentrations of 1, 10, and 100 *μ*mol/L significantly decreased PKC activity to 87.7 ± 4.2%, 74.8 ± 6.3%, and 46.3 ± 1.8%, respectively. On the other hand, HHDC did not significantly influence PKC activity (Figure 5). Since the PKC activity assay determined all PKC isoforms (*α*, *β*, *γ*, *δ*, *ε*, *μ*, *ζ*, and *θ*), we further specified the effect of HHC and HHDC on phosphorylation of PKC*α*, *β*II, and *δ*.

**Figure 6:**
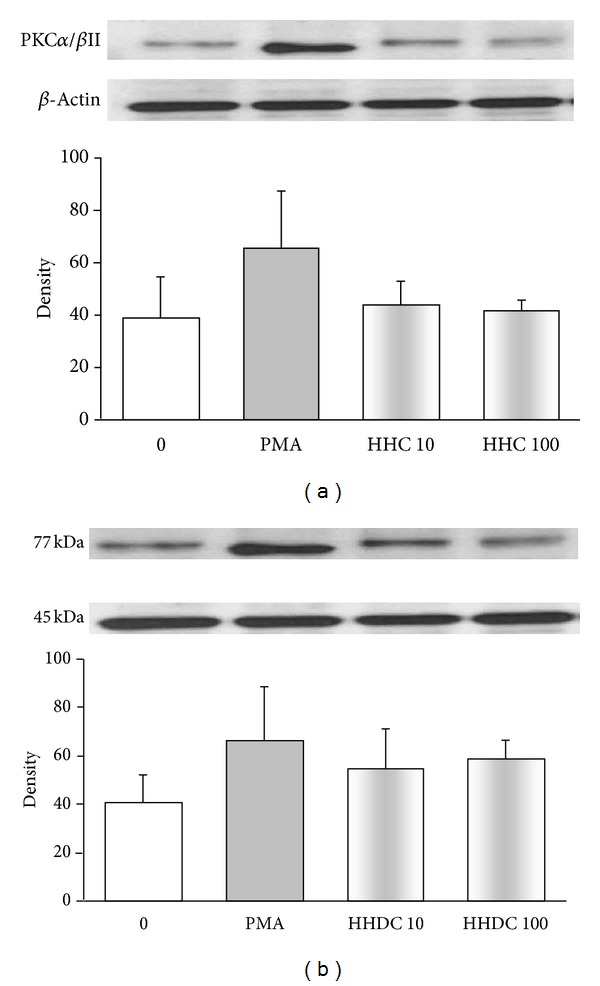
PKC phosphorylation in PMA stimulated human neutrophils treated with 10 and 100 *μ*mol/L HHC or HHDC. Human isolated neutrophils (5 × 10^6^ cells) were incubated at 37°C with HHC or HHDC (10 or 100 *μ*mol/L) for 1 min, prior to addition of PMA (0.15 *μ*mol/L). Cell lysates were prepared, and the protein levels of PKC isoenzymes (*α* and *β*II) were analyzed by Western blotting and detected by Phospho-PKC*α*/*β*II (Thr638/641) Antibody. The data are evaluated as optical density of PKC corrected to optical density of the corresponding *β*-actin band. Mean ± SEM, *n* = 8 (the data are representative of 4 donors performed in 2 separate experiments).

**Figure 7:**
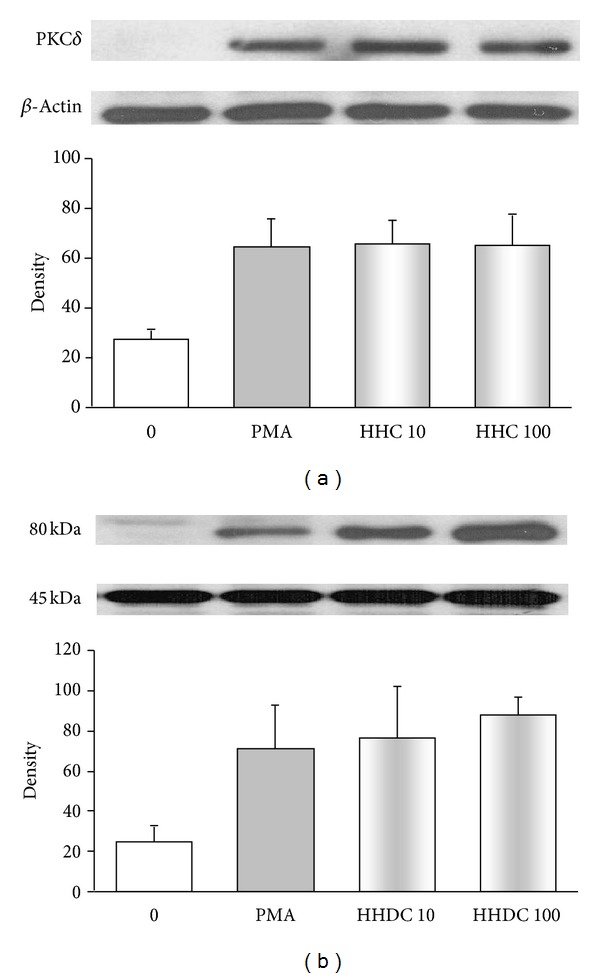
PKC phosphorylation in PMA stimulated human neutrophils treated with 10 and 100 *μ*mol/L HHC or HHDC. Human isolated neutrophils (5 × 10^6^ cells) were incubated at 37°C with HHC or HHDC (10 or 100 *μ*mol/L) for 1 min, prior to addition of PMA (0.15 *μ*mol/L). Cell lysates were prepared, and the protein level of PKC isoenzyme *δ* was analyzed by Western blotting and detected by Phospho-PKC*δ* (Thr505) Antibody. The data are evaluated as optical density of PKC corrected to optical density of the corresponding *β*-actin band. Mean ± SEM, *n* = 8 (the data are representative of 4 donors performed in 2 separate experiments).

After PMA stimulation, we observed increased phosphorylation of PKC*α*, *β*II, and *δ* in comparison with unstimulated human neutrophils. Treatment with HHC or HHDC in the concentrations of 10 and 100 *μ*mol/L resulted in decrease in the level of PKC*α*, *β*II as compared to those of control but did not affect phosphorylation of PKC*δ* (Figures 6 and 7). 

### 3.5. Effects of HHC and HHDC on the Phosphorylation of the NADPH Oxidase Component p40^*phox*^

**Figure 8:**
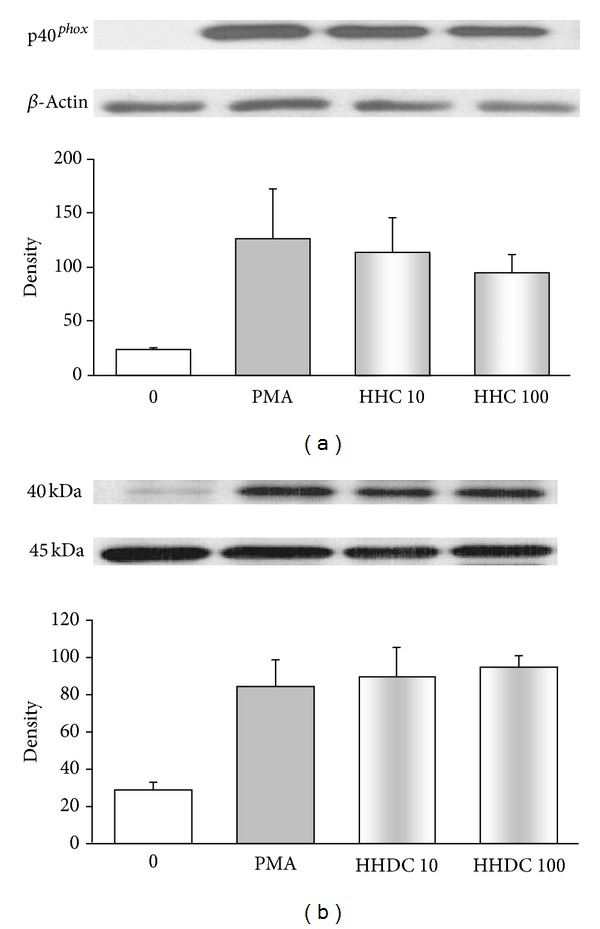
Phosphorylation of the NADPH oxidase component p40^*phox*^ in PMA stimulated human neutrophils treated with 10 and 100 *μ*mol/L HHC or HHDC. Human isolated neutrophils (5 × 10^6^ cells) were incubated at 37°C with HHC or HHDC (10 or 100 *μ*mol/L) for 1 min, prior to addition of PMA (0.15 *μ*mol/L). Cell lysates were prepared, and the protein level p40^*phox*^ subunit was analyzed by Western blotting and detected by Phospho-p40^*phox*^ (Thr 154) Antibody. The results are evaluated as optical density of p40^*phox*^ corrected to optical density of the corresponding *β*-actin band. Mean ± SEM, *n* = 8 (the data are representative of 4 donors performed in 2 separate experiments).

Recently, the selective role for p40^*phox*^ in intracellular ROS production was reported [10, 13]. We studied whether the observed inhibition of intracellular ROS production, particularly by HHC, might be connected with its effect on phosphorylation of p40^*phox*^ subunit in PMA stimulated human neutrophils. As shown in Figure 8, PMA increased phosphorylation of p40^*phox*^ subunit, yet HHC and HHDC failed to influence it.

## 4. Discussion

 Regulation of neutrophil activity is one of the important factors in achieving host defense and avoiding tissue-damaging inflammation. Recently, great attention has been devoted to polyphenols of natural origin as well as to their synthetic derivatives. Many of them were found to possess effects which could be used in prevention and support therapy of chronic inflammatory diseases [30–35]. Previously, we have suggested that different effects of phenylcoumarin derivatives HHC and HHDC on phagocyte functions may be due to their diverse free radical scavenging properties and lipophilicity features. Further, we indicated that the ability of HHC and HHDC to reduce oxidant production in neutrophils might be connected with inhibition of NADPH oxidase activity, via decrease of PKC activation [23]. In the present study we specified the site of HHC and HHDC action on ROS generation and considered some possible molecular mechanisms linking regulation of oxidant production.

 Our results showed that neutrophils activated by PMA responded by an oxidant production composed of both extra- and intracellular component of chemiluminescence. We found the extracellular ROS production to be predominant, as also demonstrated by Björnsdottir et al. [13] and Dahlgren and Karlsson [36]; however, in comparison with their results we observed a higher ratio between oxidants produced extra- and intracellularly, in favour of the former. HHC and HHDC were potent inhibitors of extracellular ROS production in human neutrophils stimulated with PMA. While HHDC reduced intracellular ROS generation only in the highest concentration tested, HHC possessed significant inhibitory effect in a concentration-dependent manner. The inhibition of both extra- and intracellular oxidant production indicated a possible interference of HHC and HHDC with ROS (scavenging activity) as well as with signalling events resulting in ROS production.

The effective inhibition of cell-free system chemiluminescence by both compounds tested (Table 2) showed that free radical scavenging properties of HHC and HHDC play an important role in the reduction of both extra- and intracellular oxidant production. The results obtained in cell-free system also suggest they interfere with peroxidase, since luminol reaction is highly dependent on the participation of myeloperoxidase. The possibility of this interaction with peroxidase is supported by findings of Kabeya et al. [37] and Andrade et al. [38] who demonstrated the inhibitory effect of 3-phenylcoumarin hydroxylated derivatives on horseradish peroxidase catalytic activity. The inhibitory effect of stilbene derivatives on myeloperoxidase release was described also by Pečivová et al. [39]. 

 The relationships between antioxidant activities and chemical structures of coumarin derivatives are important factors which may influence the oxidant production *in vitro* and *in vivo* [1, 23, 38, 40–42]. Both coumarines tested, HHC and HHDC, have a similar structure: a hydroxyl group at C7 position at ring A and a hydroxyl group at C4′ position at ring B. While HHC has an unsaturated bond between C3 and C4 at ring C, HHDC fails to possess it (Figure 1). Our results indicate that the inhibitory effect of HHC and HHDC on PMA stimulated extracellular ROS generation in neutrophils is probably not dependent on the presence of a double bond between C3 and C4 at ring C. On the other hand, the more pronounced inhibition of intracellular ROS production by HHC indicates the requirement of an unsaturated 3-, 4-binding site at ring C for this inhibitory effect. 

Polyphenols were reported to affect cell functions by modifying plasma membrane structure and physical characteristics, such as fluidity and electrical properties. These effects can be observed both when polyphenols are adsorbed on the membrane and when they are inserted into the bilayer [43]. The low effect of HHDC on intracellular ROS generation might be explained by the lower values of the partition coefficient of HHDC compared to that of HHC and thus the more efficient penetration of HHC into the membrane than that of the less lipophilic HHDC, as shown by our earlier published findings [23].

 In biological systems, ROS are generated by a number of enzymatic systems, and modifications of plasma membrane structure can result in functional changes, including the activity of membrane-associated enzymes and the modulation of signal transduction [7, 43]. 

Inhibition of PKC or downregulation of its intracellular expression and activity has been proposed as an important mechanism of the antioxidant effect of polyphenols [7, 27, 35, 44, 45]. We found that HHC, unlike HHDC, effectively decreased intracellular oxidant production involved in the regulation of neutrophil function, and we thus supposed its interaction to occur at PKC level. Since we observed a significant reduction of PKC activity by HHC, we further specified its effect on PKC*α*, *β*II phosphorylation. Inhibition of PKC activity and PKC*α*, *β*II phosphorylation by HHC only, indicates that the presence of the double bond between C3 and C4 at ring C is necessary for this effect. Structure dependence of the inhibitory effect of polyphenolic antioxidants on signal transduction enzymes, such as PKC, was found also by Varga et al. [40]. Although a specific role for PKC*δ* in the PMA-dependent intracellular activation process was reported [22], we did not observe any effect of coumarines tested on phosphorylation of PKC*δ*. 

 One of the cytosolic components of the NADPH oxidase, p40^*phox*^, which is specifically translocated to intracellular phagosomal and granule membranes, was also indicated as a further determining factor for intracellular ROS production [10, 12]. Moreover, a reduction of p40^*phox*^ phosphorylation by PKC inhibitors was found by Bouin et al. [46] and Someya et al. [47]. Our results, however, showed that in PMA stimulated neutrophils reduction of intracellular ROS generation and PKC activity by HHC was not associated with inhibition of phosphorylation of the NADPH oxidase subunit p40^*phox*^.

## 5. Conclusion

We extended our previous findings about the actions of the coumarine derivatives HHC and HHDC on the activity of human neutrophils by investigating the influence of extra- and intracellular ROS formation and some possible molecular mechanisms affecting the regulation of oxidant production. The presented results suggest that, in pathological processes in which neutrophils are involved, HHDC may act predominantly as a potent inhibitor/scavenger of extracellularly produced ROS. HHC may act both extra- and intracellularly, and, besides its direct interference with ROS, it may interfere also with the PKC signalling pathway. These findings confirmed our previous assumption that the different effects of the coumarine derivatives tested might be due to their diverse molecular structures, which provides them with different physicochemical and free radical scavenging characteristics.

## Figures and Tables

**Table 1:** Cytotoxic effect of HHC and HHDC evaluated by means of ATP liberation.

Concentration (*μ*mol/L)	ATP liberation (nmol/L)	
	HHC	HHDC
0	13.53 ± 1.59	12.38 ± 1.22
1	12.63 ± 1.67	11.99 ± 1.24
10	10.25 ± 0.37	12.57 ± 1.14
100	7.91 ± 0.41	12.94 ± 1.01

The data express ATP liberation from 30,000 neutrophils.

0: untreated neutrophils, 1–100 *μ*mol/L: treated by HHC or HHDC, total (159 ± 12 nmol/L) represents ATP content as determined immediately after complete neutrophil destruction. Mean ± SEM, *n* = 6.

**Table 2:** Doses of the compounds tested producing 50% inhibition of control extracellular CL and intracellular CL of human neutrophils and cell-free CL system.

Chemiluminescence	HHC	HHDC
	EC_50_ (*μ*mol/L)	EC_50_ (*μ*mol/L)
Extracellular	1.04 ± 0.20	1.01 ± 0.13
Intracellular	5.24 ± 0.57	>100
Cell free	0.59 ± 0.01	2.47 ± 0.04

Human isolated neutrophils (5 × 10^5^/sample) were stimulated with PMA (0.05 *μ*mol/L) at 37°C, in the presence of HHC or HHDC (0.01–100 *μ*mol/L). Extra- and intracellular ROS production were measured by isoluminol-/luminol-enhanced CL. In the cell-free system (luminol, horseradish peroxidase, and hydrogen peroxide), CL responses were measured in the presence of HHC or HHDC (1, 10, or 100 *μ*mol/L) at 37°C. Percentage inhibition was calculated on the basis of integrated values of CL over 1800 s (extra- and intracellular CL) and of cell-free CL over 10 minutes. Mean ± SEM, *n* = 3–8. Control values given in RLU × seconds were 5.8 × 10^6^ ± 1.4 × 10^6^ for extracellular CL, 5 × 10^5^ ± 0.5 × 10^4^ for intracellular CL, and 3.8 × 10^5^ ± 0.18 × 10^4^ for cell-free CL.
